# Predicting FOXM1-Mediated Gene Regulation through the Analysis of Genome-Wide FOXM1 Binding Sites in MCF-7, K562, SK-N-SH, GM12878 and ECC-1 Cell Lines

**DOI:** 10.3390/ijms21176141

**Published:** 2020-08-26

**Authors:** Keunsoo Kang, Yoonjung Choi, Hoo Hyun Kim, Kyung Hyun Yoo, Sungryul Yu

**Affiliations:** 1 Department of Microbiology, College of Science & Technology, Dankook University, Cheonan 31116, Korea; rlagngus@gmail.com; 2 Deargen Inc., Daejeon 34051, Korea; yoonjungc@deargen.me; 3 Laboratory of Biomedical Genomics, Department of Biological Sciences, Sookmyung Women’s University, Seoul 04310, Korea; khryu@sookmyung.ac.kr; 4 Research Institute of Women’s Health, Sookmyung Women’s University, Seoul 04310, Korea; 5 Department of Clinical Laboratory Science, Semyung University, Jecheon 27136, Korea

**Keywords:** FOXM1, NFY, ChIP-seq, cell cycle, master regulator, breast cancer, MCF-7

## Abstract

Forkhead box protein M1 (FOXM1) is a key transcription factor (TF) that regulates a common set of genes related to the cell cycle in various cell types. However, the mechanism by which FOXM1 controls the common gene set in different cellular contexts is unclear. In this study, a comprehensive meta-analysis of genome-wide FOXM1 binding sites in ECC-1, GM12878, K562, MCF-7, and SK-N-SH cell lines was conducted to predict FOXM1-driven gene regulation. Consistent with previous studies, different TF binding motifs were identified at FOXM1 binding sites, while the NFY binding motif was found at 81% of common FOXM1 binding sites in promoters of cell cycle-related genes. The results indicated that FOXM1 might control the gene set through interaction with the NFY proteins, while cell type-specific genes were predicted to be regulated by enhancers with FOXM1 and cell type-specific TFs. We also found that the high expression level of FOXM1 was significantly associated with poor prognosis in nine types of cancer. Overall, these results suggest that FOXM1 is predicted to function as a master regulator of the cell cycle through the interaction of NFY-family proteins, and therefore the inhibition of FOXM1 could be an attractive strategy for cancer therapy.

## 1. Introduction

The forkhead (FKH) box protein M1 (FOXM1) belongs to the forkhead transcription factor family, which plays a key role in cell cycle progression, including the G1/S phase transition and progression into mitosis [1,2]. Emerging evidence suggests that aberrant activation of FOXM1 signaling stimulates tumorigenesis and tumor aggressiveness in various cancer cells by increasing drug resistance and migration/invasion [3,4]. Consistent with its role in tumor progression, overexpression of FOXM1 has been shown to correlate with a poor prognosis in many cancer patients, including breast cancer [5,6,7,8]. In particular, recent studies have revealed that FOXM1 is tightly linked with estrogen receptor (ERα) activity and HER2 protein status in breast cancer [5,9]. Approximately 60~80% of all breast cancers are ERα positive, but only ~70% of these patients respond to endocrine treatment [10]. The remaining patients acquire resistance to this type of therapy, which is known to be mediated by a positive feedback regulatory loop between FOXM1 and ERα [11]. Indeed, inhibition of FOXM1 activity using RNA interference system and chemical compounds displayed reduced tumor growth and invasiveness, as well as increased chemosensitivity [12,13,14]. These results underline that FOXM1 is a promising target for anticancer therapy.

FOXM1 regulates the transcription of target genes by binding to a DNA sequence containing a canonical FKH motif (RYAAAYA), through a conserved ‘wing helix’ DNA binding motif [15]. Motif enrichment analysis of FOXM1-bound genes revealed that FOXM1 shares binding motifs with other forkhead family members, leucine zippers, GATA, and estrogen receptor families [9]. In the late G2/M phase, FOXM1 binds to the CHR (cell cycle genes homology region) elements and NFY (nuclear transcription factor Y) box, but not to a canonical FKH motif, suggesting that FOXM1 possibly interacts with different co-factors in various cellular environments [16]. Consistent with this, FOXM1 has discrete genomic distribution in different cell types. In the ERα-positive cancer cell line, FOXM1 simultaneously occupies the same genomic position with the estrogen receptor in an ERα-dependent manner. On the other hand, in the ERα-negative cancer cell line, FOXM1 binds mainly to the motifs of other transcription factors, such as leucine zippers and c-MYC, but not to ERα. In particular, in U2OS cells, FOXM1 exhibits a high level of concordance with the NFY CCAAT-binding motif [8,16,17]. Moreover, the FOXM1 DNA-binding domain (DBD) mutant still binds to the CCAAT-box motif via functional protein–protein interaction, despite complete loss of canonical FKH motif binding [17], suggesting that the mechanism of action of FOXM1 in transcriptional regulation of target genes is determined by cellular contexts. Interestingly, promoter elements of genes related to G2/M late cell cycles commonly contain CHR, CDE (cell cycle-dependent element) motifs, and CCAAT boxes, which are associated with the NFY transcription factor, but relatively lack the FKH motif. Nevertheless, FOXM1 controls a set of genes that are expressed during late G2/M phase, possibly through the interaction with the MMB transcriptional regulatory complex [16,18]. Conversely, FOXM1 is a putative target of the cis-regulatory module CHR/NF-Y, which represents a positive feedback regulatory loop between these proteins in the transcription of cell cycle-related genes [19].

Given that various binding motifs were identified at genome-wide FOXM1 binding sites in different cell types using a high-throughput sequencing technique (chromatin immunoprecipitation followed by sequencing; ChIP-seq) [16,20], it is of particular interest how FOXM1 simultaneously controls not only a common gene set such as cell cycle- and/or mitosis-related genes, but also cell type-specific gene sets in different cellular contexts. To this end, we characterized FOXM1-associated cis-regulatory elements through a comprehensive meta-analysis of available high-quality FOXM1 ChIP-seqs performed in ECC-1, GM12878, K562, MCF-7, and SK-N-SH cell lines from the encyclopedia of DNA elements (ENCODE) database (https://www.encodeproject.org/) [21]. Our bioinformatic analysis revealed that FOXM1 regulates a common gene set consisting of cell cycle- and/or mitosis-related genes through an interaction with NFY, while cell type-specific genes are controlled by FOXM1, possibly through the interaction with cell type-specific transcription factors and super-enhancers (SEs). Intriguingly, FOXM1 was predicted to regulate cell type-specific genes including ESR1 in the ER+ breast cancer cell line MCF-7 by directly binding to cis-regulatory elements that include the canonical FKH motif. Overall, our results suggest that FOXM1 is a master regulator of cell cycle- and/or mitosis-related genes throughout the NFY family of proteins, and that cell type-specific gene sets are controlled by the interaction between cell type-specific transcription factors and FOXM1.

## 2. Results

### 2.1. Canonical and non-Canonical FOXM1 Binding Motifs

**Figure 1:**
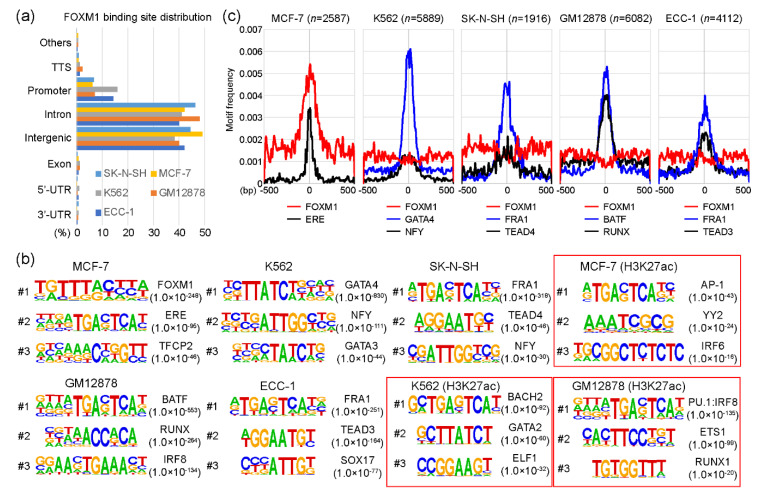
FOXM1 (Forkhead box protein M1) binding motif analysis. (**a**) Distributions of FOXM1 binding sites in SK-N-SH, MCF-7, K562, GM12878, and ECC-1 cell lines are shown, according to transcription termination site (TTS), promoter (+/− 2kb of the transcription start site), intron, intergenic, exon, 5′-unstranslated region (5′-UTR), and 3′-UTR. (**b**) Top three motifs identified with FOXM1 binding sites in a given cell line are shown. H3K27ac-enriched regions were used as control sets for the motif analysis of MCF-7, K562, and GM12878 cell lines (red boxes). (**c**) Motif frequencies of the top two motifs in each cell line were plotted according to the center of FOXM1 binding peaks.

As a transcription factor, FOXM1 binds to various target sites on the genome. Interestingly, a previous study [17] showed that the DNA binding-deficient FOXM1 mutant protein was able to bind to designated target sites through co-factor interactions without affecting the function of FOXM1. To understand the molecular mechanism underlying this characteristic, we reanalyzed FOXM1 ChIP-seq data sets performed in ECC-1, GM12878, K562, MCF-7, and SK-N-SH cell lines from the encyclopedia of DNA elements (ENCODE) website (https://www.encodeproject.org/) [21]. Each FOXM1 ChIP-seq sample was analyzed with a corresponding control sample using HOMER (Table S1). Totals of 4112, 6082, 5889, 2587, and 1916 FOXM1 binding sites (peaks) were identified in ECC-1, GM12878, K562, MCF-7, and SK-N-SH cell lines, respectively (Table S2). To gain insights into the genomic target sites of the FOXM1 transcription factor among the cell lines, we conducted a series of the following bioinformatic analyses. First, the distribution of FOXM1 binding sites on the human genome in those cell lines was investigated according to the annotation of promoters, introns, exons, transcription termination sites (TTSs), as well as 5′-UTR and 3′-UTR regions. The result indicates that most FOXM1 binding sites are located in intron and intergenic regions, followed by promoter, TTS, exon, 5′-UTR, and 3′-UTR (Figure 1a). The distributions of FOXM1 binding sites were all similar in the five cell lines. For example, 1269 (49.1%) and 1088 (42.1%) FOXM1 binding sites were located in intergenic and intron regions, respectively, while only 163 (6.3%) FOXM1 peaks were detected in promoter regions in the MCF-7 cell line. This suggests that FOXM1 acts more strongly on enhancers than on promoters. Second, we performed a series of motif analyses to characterize whether motifs identified in FOXM1 binding sites varied among cell lines. This is a possible mode of action, because a previous report showed that the function of FOXM1 was not significantly affected in FOXM1 mutants with an almost complete loss of DNA-binding [17]. Interestingly, we found that motifs identified in FOXM1 binding sites were indeed different among cell lines, indicating that FOXM1 is not directly recruited to chromatin, but is related through co-factor interactions, depending on the cell type. For example, among five cell lines, the canonical FOXM1 binding FKH motif was only identified in the FOXM1 binding sites in the MCF-7 cell line (*p*-value < 1.0 × 10^−248^), while GATA4 (*p*-value < 1.0 × 10^−830^), FRA1 (*p*-value < 1.0 × 10^−318^), FRA1 (*p*-value < 1.0 × 10^−251^), and BATF (*p*-value < 1.0 × 10^−553^) motifs were significantly detected in the FOXM1 binding sites of K562, SK-N-SH, ECC-1, and GM12878 cell lines, respectively (Figurfe 1b). These motifs were specific to the FOXM1 binding sites, since motifs found in H3K27ac-enriched regions (active sites) of the same cell line showed much higher *p*-values than those in the FOXM1 binding sites. To ascertain the finding, the average motif frequency for FOXM1 peaks was calculated. As expected, all top motifs were located around the center of FOXM1 binding sites, and the most significantly associated motifs were in general located at the center of the FOXM1 binding sites (Figure 1c). Similar to the above result, the canonical FOXM1 binding motif was only significantly observed near the center of FOXM1 binding sites in the MCF-7 cell line, while it was not associated with FOXM1 binding sites in the other cell lines. Overall, these results suggest that FOXM1 is recruited to target sites either by recognizing the canonical binding motif or through co-factor interactions, depending on cellular contexts.

### 2.2. Functional Prediction of FOXM1 Target Genes

**Figure 2:**
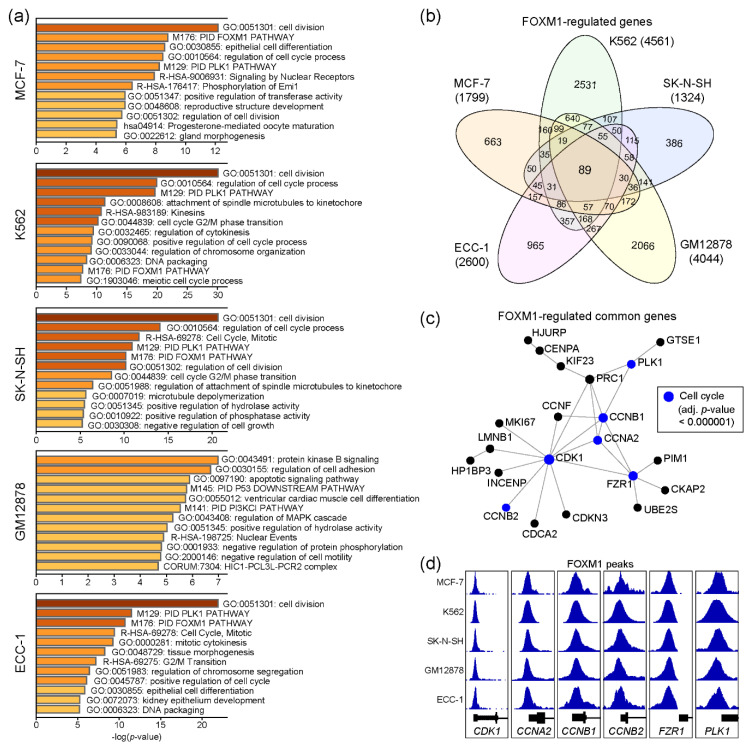
Molecular pathway prediction of FOXM1 target genes. (**a**) Gene ontology (GO) analysis was conducted using Metascape with the top 500 genes (ranked by the height of FOXM1 peaks) around the FOXM1 binding sites. (**b**) A Venn diagram shows the number of FOXM1 target genes identified in five cell lines. (**c**) A protein–protein interaction network was constructed with 89 common FOXM1-target genes among cell lines using NetworkAnalyst. (**d**) FOXM1 binding at promoters of the CDK1, CCNA2, CCNB1, CCNB2, FZR1, and PLK1 genes are shown.

FOXM1 regulates several important biological pathways, including cell cycles [16]. To characterize functions of the FOXM1 transcription factor in each cell line, we performed gene ontology (GO) analysis with the genes nearby FOXM1 binding sites, using Metascape [22]. Since the number of genes may affect the result, we only used the top 500 FOXM1 binding sites, sorted by the density of FOXM1 ChIP-seq reads (descending order) in each cell line (Table S2). Interestingly, the result indicates that these FOXM1 target genes are significantly associated with cell cycle-related pathways in the MCF-7, K562, SK-N-SH, and ECC-1 cell lines, except for GM12878 (Figure 2a). To further dissect the roles of the identified FOXM1 binding sites, in terms of regulatory elements (promoter vs enhancer), FOXM1 binding sites were classified into either promoter-associated (+/− 2kb of the transcription start site) or enhancer-associated groups, depending on their locations from the transcription start sites of known genes (Figure S1a). The results indicated that the FOXM1 binding sites with the enhancer regions were clearly associated with the enhancer marks (H3K4me1 and/or H3K27ac) [23], implying the role of FOXM1 through enhancer elements for gene regulation. In addition, genes associated with FOXM1 binding sites within their promoter regions were predicted to be involved in cell cycle-related pathways in all five cell lines, while enhancer-associated FOXM1 binding sites were related to cell type-specific biological pathways (Figure S1b), as reported previously [24]. Next, comparison of the FOXM1 target genes between cell lines revealed that 89 genes were common across all five cell lines (Table S3), while many target genes were unique to each cell line (Figure 2b). Protein–protein interaction (PPI)-based network analysis for the 89 genes further identified a core PPI network, including CDK1, CCNB2, CCNA2, CCNB1, PLK1, and FZR1 (Figure 2c). These are key proteins known to regulate cell cycles [25,26]. Although most FOXM1 binding sites were located in intron and intergenic regions (Figure 1a), intriguingly, the promoters of these genes were strongly occupied by FOXM1 in all five cell lines (Figure 2d). Thus, this result suggests that one of key functions of FOXM1 is to control expression levels of cell cycle-related genes, regardless of most, if not all, cell types.

### 2.3. The association of FOXM1 and NFY in Regulating Cell-Cycle-Related Genes

**Figure 3:**
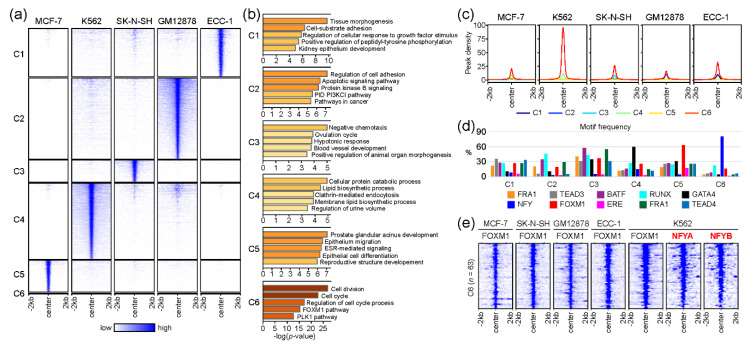
Comparative analysis of FOXM1 binding sites between cell lines. (**a**) The heatmap shows common (C6) and unique (C1–C5) clusters of FOXM1 binding sites between MCF-7, K562, SK-N-SH, GM12878, and ECC-1 cell lines. (**b**) Gene ontology (GO) analysis was performed using Metascape with the top 500 genes (ranked by the height of FOXM1 peaks) around the FOXM1 binding sites in each cluster. (**c**) The line plots show the average of the FOXM1 peak signal at the FOXM1 binding sites. (**d**) The bar plot shows the frequency of motif counts found at the FOXM1 binding sites of the clusters. (**e**) The heatmap shows binding profiles of given transcription factors on FOXM1 binding sites in the C6 cluster. NFYA and NFYB ChIP-seq were reanalyzed from a previous study (GSE26439) [29].

Although we discovered a core gene set likely to be regulated by FOXM1 across five cell lines in the gene-based comparison (Figure 2), we further investigated the molecular mechanism of FOXM1 by comparing FOXM1 binding sites (peak-centered) between cell lines. This peak-based approach clearly identified FOXM1 binding sites unique to each cell line, while discovering common FOXM1 binding sites (Figure 3a). The list of all FOXM1 peaks in each cluster is provided in Table S4. Interestingly, a small number (*n* = 63) of FOXM1 binding sites in a cluster (denoted as C6) were found in all cell lines (Figure 3a), while the majority of FOXM1 peaks were unique to each cell type. Similar to the gene-based approach (Figure 2), gene ontology analysis of the genes near the common 63 FOXM1-binding sites reveals that cell cycle-related pathways (cell division, cell cycle, regulation of cell cycle process, FOXM1 pathway, and the PLK1 pathway) are significantly associated with these FOXM1-binding sites. On the other hand, the rest of the clusters (C1–C5) contained FOXM1 peaks unique to each cell line, highlighting that cell type-specific pathways are also significantly associated with FOXM1. For example, the C5 cluster, which contained FOXM1-binding sites unique to the MCF-7 cell line, is significantly related to the ESR-mediated signaling pathway. The estrogen receptor 1 (ESR1) gene encodes an estrogen receptor that localizes to the nucleus upon the binding of 17β-estradiol (E2) at the membrane [27], and is therefore a key protein in the pathway. In addition, the average enrichment level (peak density) of the FOXM1 occupancy was the highest on the C6 cluster (Figure 3c), suggesting that cell cycle-related pathways are the main target of the FOXM1 protein in most, if not all, cell types. Next, we further characterized the FOXM1 binding sites in each cluster, by means of the motifs identified in the previous analysis (Figure 1b). Similar motif frequencies were found in each cell type-specific cluster (C1–C5, Figure 3d), while 81% (51 out of 63) FOXM1 binding sites in the common C6 cluster included NFY binding motifs. In addition, 92% (58 out of 63) of C6-FOXM1 binding sites were located within 2kb of the transcription start site (TSS) of the target genes (Table S4). This indicates that FOXM1 potentially regulates cell cycle-related genes by binding to their promoters through NFY complexes. The NFY complex consists of three different subunits: NFYA, NFYB, and NFYC. Among them, NFYA only contains a DNA-binding domain, and therefore is responsible for recognizing the CCAAT motif box (NFY binding motif) [28]. To ascertain the prediction based on the motif analysis, we reanalyzed NFYA and NFYB ChIP-seq performed in K562 cells from a previous study [29]. Surprisingly, most of the FOXM1 binding sites in the C6 cluster coincided with NFYA and NFYB (Figure 3e). This finding suggests that NFY family proteins are directly bound to these target sites, while FOXM1 is tethered to these sites through interactions with NFY family proteins. Overall, these results indicate that the main role of the FOXM1 protein is to control the transcription of genes involved in cell cycle-related pathways, through the interaction with NFY family proteins. However, further experimental validation is needed.

### 2.4. FOXM1 and Super-Enhancers

**Figure 4:**
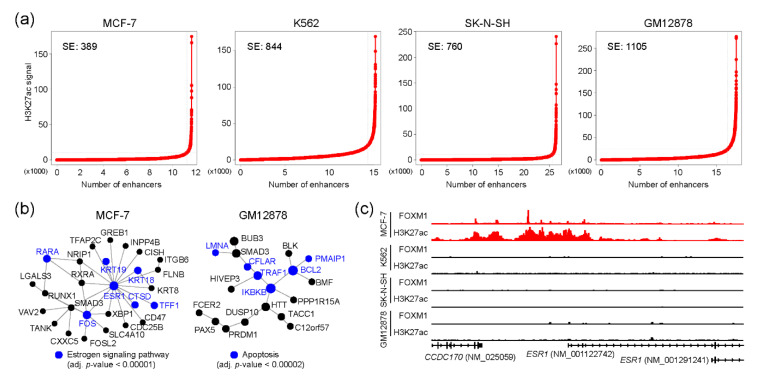
FOXM1 binding sites associated with super-enhancers. (**a**) Super-enhancer line plots indicate the number of identified super-enhancers using H3K27ac-enriched regions. (**b**) Protein–protein interaction networks were constructed using NetworkAnalyst. Genes around the top 300 FOXM1 binding sites (ranked by the height of the peaks) were used for the analysis. (**c**) FOXM1 binding and H3K27ac enrichment near the *ESR1* gene is shown.

Super-enhancers (SEs) are the enhancers that contain clusters of enhancers and regulate cell-type-specific gene regulatory networks [30], and are therefore known to define cell identity and be associated with human diseases [31,32]. We wondered whether FOXM1 binding sites were also associated with SEs for controlling cell-type-specific gene regulatory programs in each cell type. To this end, SEs were identified in MCF-7, K562, SK-N-SH, and GM12878 cell lines using corresponding H3K27ac ChIP-seq with the ROSE algorithm [33]. ECC-1 was excluded from this analysis, due to the lack of a corresponding H3K27ac ChIP-seq sample in the ENCODE database. Totals of 389, 844, 760, and 1105 SEs were identified in MCF-7, K562, SK-N-SH, and GM12878 cell lines, respectively (Figure 4a and Table S5). Next, we pinpointed FOXM1 binding sites which were overlapped with SEs in each cell line. This analysis identified 401, 603, 226, and 1235 FOXM1 binding sites in MCF-7, K562, SK-N-SH, and GM12878 cell lines, respectively. To unveil potential functions underlying these FOXM1-associated SE-regulated genes, we conducted a PPI-based network analysis with the genes around the top 300 FOXM1 binding sites in each cell line. As expected, this analysis identified cell type-specific core PPI networks in the MCF-7 and GM12878 cell lines. Similar to the previous result (Figure 3b), the PPI network with proteins involved in the estrogen signaling pathway was the main PPI network in the MCF-7 cell line, and the ESR1 protein was predicted as the core protein controlling the other proteins in the network (Figure 4b). In fact, we found that a super-enhancer was located at the upstream of the ESR1 gene, and there were several FOXM1 binding sites located within the SE (Figure 4c). The results illustrate the connection between FOXM1 and SE in regulating target genes. In sum, our SE-based analysis also discovered that FOXM1 not only regulates cell cycle-related pathways, but is also responsible for cell type-specific pathways, possibly through SEs.

### 2.5. FOXM1 as a Prognostic Marker for Various Types of Cancer

**Figure 5:**
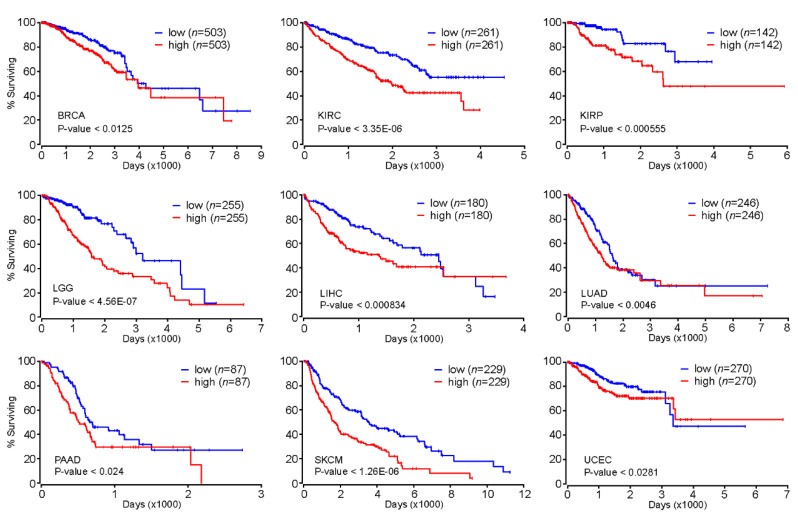
Kaplan–Meier survival analysis based on the FOXM1 expression level. Kaplan–Meier (KM) survival analysis was conducted using Oncolnc (http://www.oncolnc.org/). Patients were divided into high (50%) or low (50%) groups based on the expression level of the *FOXM1* gene.

Our comprehensive analysis of genome-wide FOXM1 binding sites revealed that FOXM1 is likely to regulate cell-cycle-related genes as well as cell-type-specific gene-regulatory network programs. These findings indicated that FOXM1 might participate in the onset and/or progression of diseases such as cancers because many cancers exhibit uncontrolled cell cycles [34]. Accordingly, we further investigated whether the expression level of *FOXM1* could be a prognostic marker for various types of cancers. Intriguingly, we found that patients with greater expression of the *FOXM1* gene significantly had significantly poorer prognoses than the other group with lesser expression of *FOXM1* in breast invasive carcinoma (BRCA), kidney renal clear-cell carcinoma (KIRC), kidney renal papillary cell carcinoma (KIRP), brain lower grade glioma (LGG), liver hepatocellular carcinoma (LIHC), lung adenocarcinoma (LUAD), pancreatic adenocarcinoma (PAAD), skin cutaneous melanoma (SKCM), and uterine corpus endometrial carcinoma (UCEC) (Figure 5). In addition, we also found significant positive correlations of gene expressions between *FOXM1* and the *NFY* subunits (*NFYA*, *NFYB* or *NFYC*) in several cancer types (Figure S2). Therefore, further experiments regarding the interaction between FOXM1 and the NFY complex are needed to demonstrate a key axis that can mitigate deregulated cell cycle-related pathways observed in various cancers.

According to our genome-wide FOXM1 binding site and motif analysis (Figure 1, Figure 2, Figure 3 and Figure 4), it is expected that available FOXM1 inhibitors could be mostly effective in the MCF-7 cell line. As predicted, a previous report [35] showed that a FOXM1 inhibitor (FDI-6) effectively showed anti-cancer effects by downregulating FOXM1-activated genes through the interruption of the DNA binding activity of the FOXM1 protein in MCF-7 cells. Overall, these results indicate that the expression level of FOXM1 can be a prognostic marker for at least nine different cancer types, and the inhibition of FOXM1 may be an attractive strategy for cancer therapy.

## 3. Discussion

FOXM1 has been known as a transcriptional factor in regulating the transcription of cell cycle-related genes [16,36,37,38]. Unlike typical transcription factors, such as STAT5A [39] and YY1 [40], various non-canonical FOXM1 binding motifs were identified depending on cell types [17]. The purpose of this study was to systematically characterize this unique feature of FOXM1, by reanalyzing FOXM1 ChIP-seq data sets in the ENCODE database. To this end, we performed a series of bioinformatic analysis based on genome-wide FOXM1 binding identified in the ECC-1, GM12878, K562, MCF-7, and SK-N-SH cell lines. Our comprehensive analysis was conducted with two different approaches, gene-based (Figure 2) and peak-based (Figure 3). Interestingly, both approaches identified a small number of common FOXM1 target genes, in which most promoters were strongly occupied by FOXM1, in all cell lines examined. Since these FOXM1 target genes were significantly associated with cell cycle-related pathways (Figure 2 and Figure 3), the result provided clear evidence that FOXM1 acts as a master regulator of the cell cycle in most, if not all, cells. This result is also supported by previous studies [15,16,36,38,41]. We also found that a number of promoters of cell cycle- and mitosis-related genes including cyclin A2 (*CCNA2*), cyclin B1 (*CCNB1*), cyclin B2 (*CCNB2*), cyclin F (*CCNF*), cell division cycle 25B (*CDC25B*), cell division cycle associated 2 (*CDCA2*), cyclin dependent kinase 1 (*CDK1*), cyclin dependent kinase inhibitor 2D (*CDKN2D*), cyclin dependent kinase inhibitor 3 (*CDKN3*), centromere protein A (*CENPA*), centromere protein F (*CENPF*), and polo like kinase 1 (*PLK1*) were strongly occupied by FOXM1 in all five cell lines (Table S4). This is a full list of FOXM1-regulated cell cycle- and/or mitosis-related genes, highlighting a single transcription factor is predicted to control a large number of known cell cycle- and mitosis-related genes across the genome. Thus, these results suggest that FOXM1 is a master regulator of the cell cycle although additional experimental validations will be required for those target genes.

The binding motif analysis revealed that various non-canonical motifs were significantly associated with cell type-specific FOXM1 binding sites, whereas the canonical FOXM1 binding motif was only detected in MCF-7 cells. This means that FOXM1 can directly bind to target sites by recognizing the known canonical ‘TAAACA’ motif [42] in the MCF-7 cell line, but not in the ECC-1, GM12878, K562, and SK-N-SH cell lines. Similarly, previous studies have shown this atypical binding mechanism of FOXM1 in vitro [16,42,43]. In addition to these non-canonical FOXM1 binding motifs, the binding motif analysis (Figure 1 and Figure 3d) also found that most of the promoter regions of the cell cycle- and mitosis-related genes (the C6 cluster, Figure 3d) were strongly occupied by FOXM1 in the ECC-1, GM12878, K562, MCF-7, and SK-N-SH cell lines. Interestingly, only 10 (16%) FOXM1 binding sites contained canonical FOXM1 motifs, while 52 (84%) FOXM1 binding regions included NFY motifs (Table S4). This unexpected frequency of NFY motifs in the strong FOXM1 binding sites illustrated that the mode of action of FOXM1 in regulating the cell cycle- and mitosis-related genes is possibly through direct or indirect interaction with NFY family proteins as a co-factor, but not through the direct DNA-binding. As predicted, we found strong NFYA and NFYB binding to the common FOXM1 binding sites (Figure 3e) in K562 cells, suggesting that NFY complexes are most likely the bridge between the FOXM1 binding sites and the FOXM1 protein. This finding is also supported by previous studies showing the interaction between FOXM1 and NFY family proteins [18,20,37,44,45,46]. We provide a full list of FOXM1 target genes including cell cycle- and mitosis-related genes as well as cell-type-specific genes with the identified motifs in Table S4.

As FOXM1 can directly bind to target sites by recognizing the canonical binding motif in the MCF-7 cell line, FOXM1 could be a key transcription factor in regulating various pathways in estrogen receptor (ER)-positive breast cancer. Interestingly, we found that several FOXM1 binding sites were located at the upstream of the ESR1 gene in a super-enhancer (Figure 4c). Protein–protein interaction-based networks with FOXM1-associated SE also predicted that ESR1 is the key protein in terms of the number of interactions (nodes) (Figure 4b). Consistent with this, the relationship between FOXM1 and ESR1 has been highlighted in previous studies. Madureira et al. showed that FOXM1 regulates the transcription of ESR1 in breast cancer cell lines [47]. Sanders et al. [9] and Wang et al. [20] found genome-wide co-binding sites of FOXM1 and ESR1 in breast cancer cell lines, similar to the result (Figure 1c). In contrast, an inverse correlation between FOXM1 and ESR1 mRNAs was also reported [48]. In addition to the previous reports, this study further revealed an additional regulatory axis of the ESR1 gene by FOXM1 and the upstream super-enhancer. This kind of regulatory axis can partially explain the resistance mechanism mediated by FOXM1 that has been observed in several cancers [49,50,51,52,53].

As a master regulator of the cell cycle, FOXM1 is highly likely to be an important transcription factor for various types of cancer. As expected, patients with higher expression levels of FOXM1 showed poor prognosis in BRCA, KIRC, KIRP, LGG, LIHC, LUAD, PAAD, SKCM, and UCEC (Figure 5), suggesting that the expression level of FOXM1 could be used as a prognostic marker for those cancer types. In addition, the concordant pattern of the FOXM1 expression emphasizes that the deregulated (most likely accelerated) cell cycle of those cancer cells partially mediated by FOXM1 is one of the key factors that must be considered when developing drugs for such cancers. Currently, several FOXM1 inhibitors, including FDI-6 [35] and RCM-1 [54], have shown anti-cancer effects through the inhibition of FOXM1 in vitro. According to the molecular mechanism proposed in this study, developing novel drugs that target FOXM1 is expected to be effective for various types of cancers, including breast cancer. Further experimental validations will shed light on unveiling the proposed FOXM1-mediated gene regulation through the NFY complex and/or cell type-specific transcription factors.

## 4. Conclusions

FOXM1 regulates common and cell-type-specific gene sets by binding to their promoters and enhancers with various transcription factors. FOXM1 is predicted to control the transcription of the common gene set, which encodes cell cycle-related proteins, through interaction with NFY proteins. As most cancer cells exhibit uncontrolled cell cycles, the inhibition of FOXM1 could be an attractive strategy for the development of anticancer drugs.

## 5. Materials and Methods

### 5.1. ChIP-seq Data Analysis

FOXM1 and H3K27ac ChIP-seq data sets performed in ECC-1, GM12878, HEK293T, K562, MCF-7, and SK-N-SH cell lines were downloaded from the encyclopedia of DNA elements (ENCODE) website (https://www.encodeproject.org/) [21]. Corresponding control ChIP-seq samples were also downloaded to remove false positive peaks. Sequencing adapter and/or raw quality portions of reads were trimmed using Trim Galore with Cutadapt (https://www.bioinformatics.babraham.ac.uk/projects/trim_galore/). Trimmed reads were aligned to the reference human genome (hg38 genome assembly) using Bowtie2 [55] with default parameters. Potential PCR-duplicates among reads were discarded using Sambamba [56]. The cleaned reads were analyzed to detect enriched regions (peaks) of a given ChIP-seq sample, using HOMER [57] with default parameters, with a false discovery rate-adjusted p-value cutoff of 0.001 (‘-style factor’ and ‘-style histone’ for transcription factors and histone marks, respectively). To get reliable binding sites of FOXM1 in each cell line, peaks that were not detected in two biological replicates were further discarded using bedtools [58].

### 5.2. Identification of Super-Enhancers

Super-enhancers (SE) were identified with H3K27ac-enriched peaks. Firstly, H3K27ac peaks were identified with their corresponding input controls using HOMER (‘-style histone’ option was used) [57]. Mapped reads of two biological replicates in each cell line were merged before the super-enhancer calling using Bamtools [59]. Then, the ranking of super enhancer (ROSE) algorithm [33] was used to define SEs with the identified H3K27ac peaks.

### 5.3. Motif Analysis

The HOMER motif analysis pipeline (http://homer.ucsd.edu/homer/ngs/peakMotifs.html) was used to find motif sequences for given peaks with the following parameters: -size 150 and -mask [57].

### 5.4. Gene Ontology, Protein–Protein Interaction-Based Network Analysis, and Visualization

Gene ontology analysis was conducted using Metascape [22] with default parameters. Protein–protein interaction (PPI) database-driven network analysis was performed using NetworkAnalyst [60] with IMEx Interactome database [61]. The zero-order network algorithm was used to construct PPI networks. Line plots and heatmaps were generated using Deeptools [62].

## Supplementary Materials

Supplementary materials can be found at https://www.mdpi.com/1422-0067/21/17/6141/s1. Table S1: List of ChIP-seq data used in this study, Table S2: FOXM1 binding sites identified in MCF-7, K562, SK-N-SH, GM12878, and ECC-1 cell lines, Table S3: A list of FOXM1-regulated common genes, Table S4: FOXM1 peak clusters (C1-C6), Table S5: FOXM1 binding sites associated with super-enhancers, Figure S1: Functional predictions of FOXM1 binding sites, Figure S2: Correlation of expression levels of the FOXM1 and NFY subunit-encoded (NFYA, NFYB, or NFYC) genes.

## Abbreviations

FKH	Forkhead
DBD	DNA binding domain
CHR	Cell cycle genes homology region
ChIP-seq	Chromatin immunoprecipitation followed by sequencing
ENCODE	The encyclopedia of DNA elements
GO	Gene ontology
PPI	Protein–protein interaction
SE	Super-enhancer
BRCA	Breast invasive carcinoma
KIRC	Kidney renal clear cell carcinoma
KIRP	Kidney renal papillary cell carcinoma
LGG	Brain lower grade glioma
LIHC	Liver hepatocellular carcinoma
LUAD	Lung adenocarcinoma
PAAD	Pancreatic adenocarcinoma
SKCM	Skin cutaneous melanoma
UCEC	Uterine corpus endometrial carcinoma
