# Supplementary material for: Predicting FOXM1-Mediated Gene Regulation through the Analysis of Genome-Wide FOXM1 Binding Sites in MCF-7, K562, SK-N-SH, GM12878 and ECC-1 Cell Lines
Source: Int J Mol Sci. 2020 Aug 26;21(17):6141. doi: 10.3390/ijms21176141 (PMC7503762; doi:10.3390/ijms21176141)
Supplement: Supplementary file 1 [file ijms-21-06141-s001.zip › Supplementary_Figures.pdf]

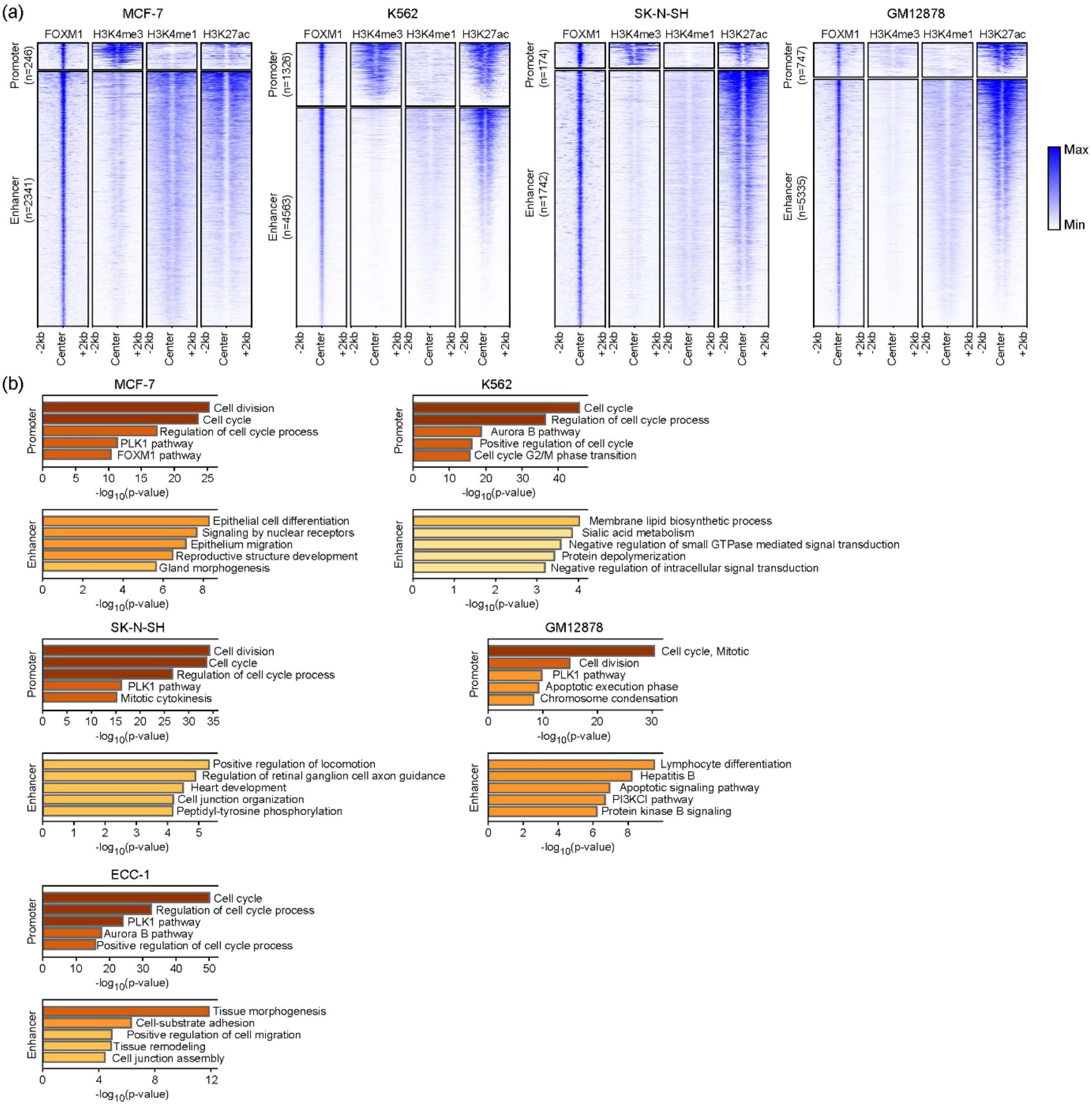

Figure S1. Functional predictions of FOXM1 binding sites. (a) Heatmaps show patterns of active histone marks (H3K4me3, H3K4me1, and H3K27ac) associated with FOXM1 binding sites in promoter (+/- 2kb of the transcription start site) and enhancer regions. Each horizontal line in the heatmaps represents a single FOXM1 binding site. The y-axis was scaled to the same length. (b) Gene ontology analysis predicted functions of FOXM1 binding sites in promoter or enhancer regions. The top 500 FOXM1 binding sites in each category were analyzed using Metascape.

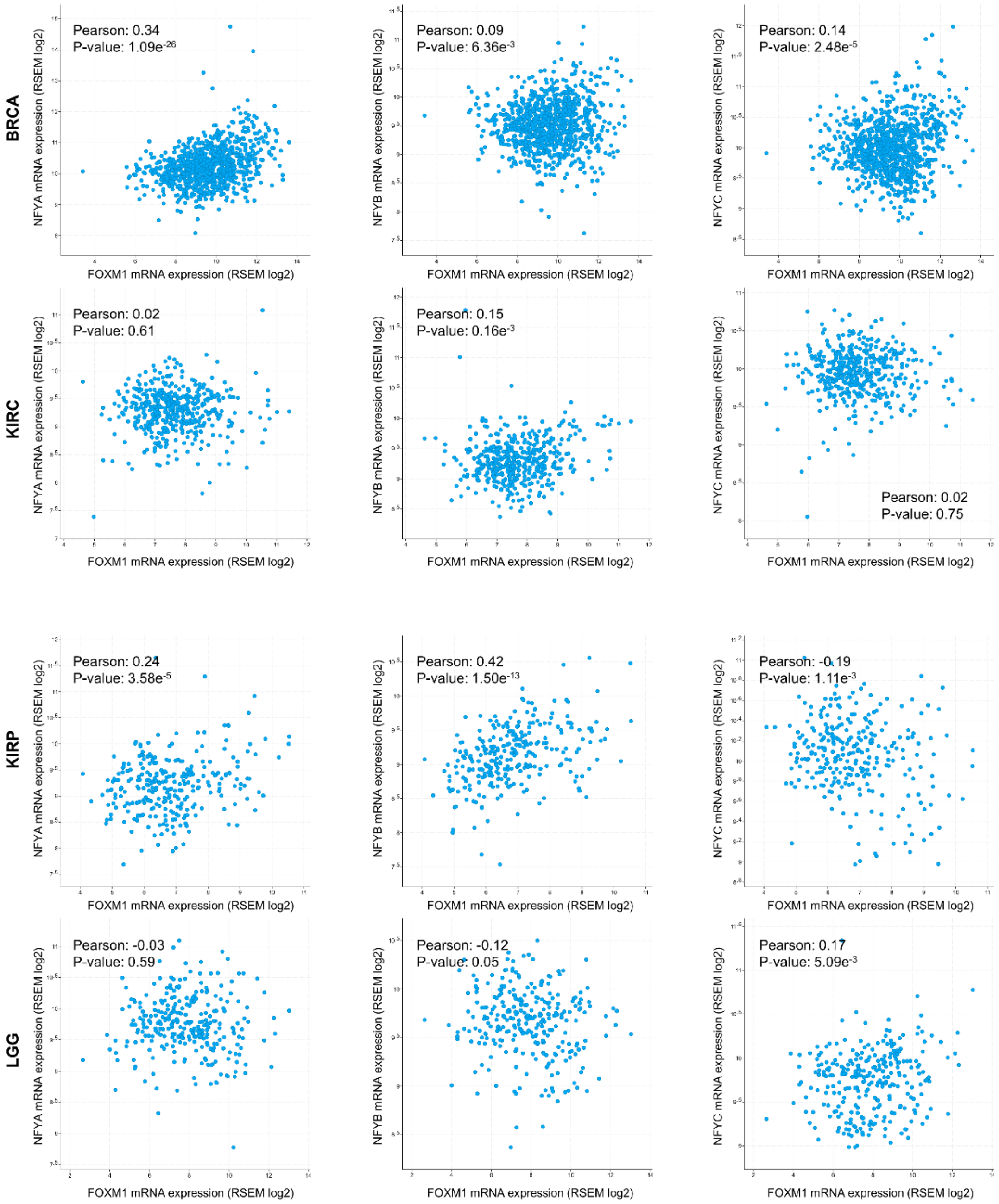
BRCA
Pearson: 0.34
P-value: 1.09e-26
NFYA mRNA expression (RSEM log2)
FOXM1 mRNA expression (RSEM log2)
Pearson: 0.09
P-value: 6.36e-3
NFYB mRNA expression (RSEM log2)
FOXM1 mRNA expression (RSEM log2)
Pearson: 0.14
P-value: 2.48e-5
NFYC mRNA expression (RSEM log2)
FOXM1 mRNA expression (RSEM log2)
KIRC
Pearson: 0.02
P-value: 0.61
NFYA mRNA expression (RSEM log2)
FOXM1 mRNA expression (RSEM log2)
Pearson: 0.15
P-value: 0.16e-3
NFYB mRNA expression (RSEM log2)
FOXM1 mRNA expression (RSEM log2)
Pearson: 0.02
P-value: 0.75
NFYC mRNA expression (RSEM log2)
FOXM1 mRNA expression (RSEM log2)
KIRP
Pearson: 0.24
P-value: 3.58e-5
NFYA mRNA expression (RSEM log2)
FOXM1 mRNA expression (RSEM log2)
Pearson: 0.42
P-value: 1.50e-13
NFYB mRNA expression (RSEM log2)
FOXM1 mRNA expression (RSEM log2)
Pearson: -0.19
P-value: 1.11e-3
NFYC mRNA expression (RSEM log2)
FOXM1 mRNA expression (RSEM log2)
LGG
Pearson: -0.03
P-value: 0.59
NFYA mRNA expression (RSEM log2)
FOXM1 mRNA expression (RSEM log2)
Pearson: -0.12
P-value: 0.05
NFYB mRNA expression (RSEM log2)
FOXM1 mRNA expression (RSEM log2)
Pearson: 0.17
P-value: 5.09e-3
NFYC mRNA expression (RSEM log2)
FOXM1 mRNA expression (RSEM log2)

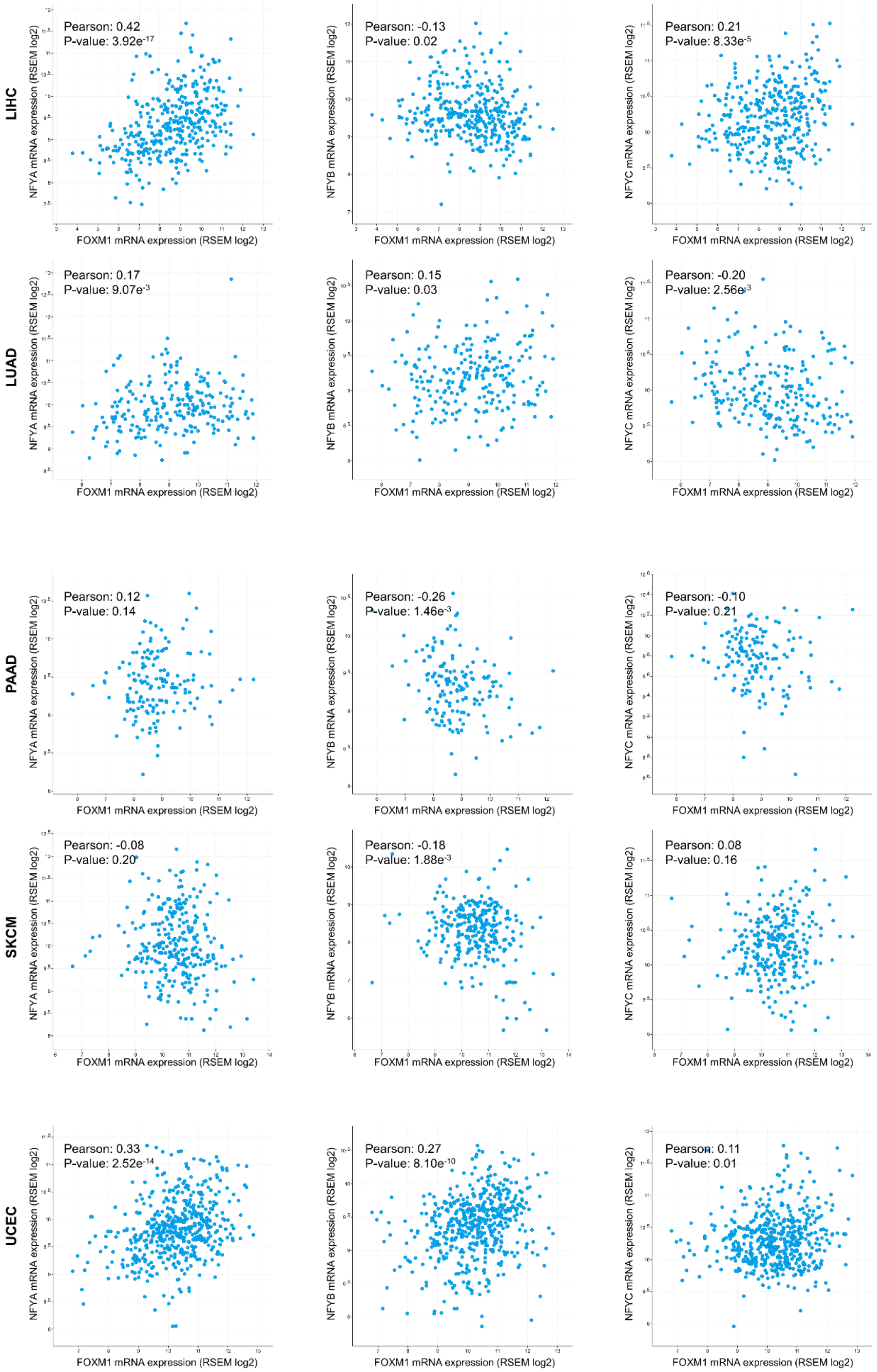

Figure S2. Correlation of expression levels of the *FOXM1* and NFY subunit-encoded (*NFYA*, *NFYB*, or *NFYC*) genes. Normalized expression levels of given genes in patients (blue dot) were plotted in scatter

plots, and Pearsons' correlation coefficients (Pearson) were calculated. The analysis was conducted using the cBioPortal application (https://www.cbioportal.org/). BRCA, breast invasive carcinoma; KIRC, kidney renal clear cell carcinoma; KIRP, kidney renal papillary cell carcinoma; LGG, brain lower grade glioma; LIHC, liver hepatocellular carcinoma; LUAD, lung adenocarcinoma; PAAD, pancreatic adenocarcinoma; SKCM, skin cutaneous melanoma; UCEC, uterine corpus endometrial carcinoma.
